# Integrated, cross-sectoral psycho-oncology (isPO): a new form of care for newly diagnosed cancer patients in Germany

**DOI:** 10.1186/s12913-022-07782-0

**Published:** 2022-04-22

**Authors:** Michael Kusch, Hildegard Labouvie, Vera Schiewer, Natalie Talalaev, Jan C. Cwik, Sonja Bussmann, Lusine Vaganian, Alexander L. Gerlach, Antje Dresen, Natalia Cecon, Sandra Salm, Theresia Krieger, Holger Pfaff, Clarissa Lemmen, Lisa Derendorf, Stephanie Stock, Christina Samel, Anna Hagemeier, Martin Hellmich, Bernd Leicher, Gregor Hültenschmidt, Jessica Swoboda, Peter Haas, Anna Arning, Andrea Göttel, Kathrin Schwickerath, Ullrich Graeven, Stefanie Houwaart, Hedy Kerek-Bodden, Steffen Krebs, Christiana Muth, Christina Hecker, Marcel Reiser, Cornelia Mauch, Jennifer Benner, Gerdamarie Schmidt, Christiane Karlowsky, Gisela Vimalanandan, Lukas Matyschik, Lars Galonska, Annette Francke, Karin Osborne, Ursula Nestle, Markus Bäumer, Kordula Schmitz, Jürgen Wolf, Michael Hallek

**Affiliations:** 1 Department of Internal Medicine I, Faculty of Medicine, Cologne University Hospital, University of Cologne, Cologne, Germany; 2 Department of Clinical Psychology and Psychotherapy, Faculty of Human Sciences, University of Cologne, Cologne, Germany; 3 University of Cologne, Faculty of Medicine and University Hospital Cologne, Institute of Medical Sociology Health Services Research, and Rehabilitation Science, Cologne, Germany; 4 University of Cologne, Faculty of Human Sciences & Faculty of Medicine and University Hospital Cologne, Institute of Medical Sociology Health Services Research, and Rehabilitation Science, Cologne, Germany; 5 Institute for Health Economics and Clinical Epidemiology (IGKE), Faculty of Medicine, University of Cologne, University Hospital Cologne, Cologne, Germany; 6 Institute of Medical Statistics and Computational Biology, Faculty of Medicine, University of Cologne, Cologne, Germany; 7 Department of Computer Science (Medical Informatics), University of Applied Sciences and Arts Dortmund, Dortmund, Germany; 8 Krebsgesellschaft Nordrhein-Westfalen E.V, Düsseldorf, Germany; 9 House of the Cancer Patient Support Associations of Germany, Bonn, Germany; 10 hautarztpraxis-wiener-platz.de, Cologne, Germany; 11 PIOH Köln - Praxis Internistischer Onkologie Und Hämatologie, Cologne, Germany; 12 GFO Kliniken Troisdorf, Troisdorf, Germany; 13 Johanna-Etienne-Krankenhaus gGmbH, Neuss, Germany; 14 Kliniken Maria Hilf GmbH, Mönchengladbach, Germany

**Keywords:** Psycho-oncology, Cancer, Practice-based research, Implementation, Dissemination, Stepped-care, Complex intervention, Quality improvement

## Abstract

**Background:**

The annual incidence of new cancer cases has been increasing worldwide for many years, and is likely to continue to rise. In Germany, the number of new cancer cases is expected to increase by 20% until 2030. Half of all cancer patients experience significant emotional and psychosocial distress along the continuum of their disease, treatment, and aftercare, and also as long-term survivors. Consequently, in many countries, psycho-oncological programs have been developed to address this added burden at both the individual and population level. These programs promote the active engagement of patients in their cancer therapy, aftercare and survivorship planning and aim to improve the patients' quality of life.

In Germany, the *“new form of care isPO”* (“nFC-isPO”; integrated, cross-sectoral psycho-oncology/**i**ntegrierte, **s**ektorenübergreifende Psycho-Onkologie) is currently being developed, implemented and evaluated. This approach strives to accomplish the goals devised in the National Cancer Plan by providing psycho-oncological care to all cancer patients according to their individual healthcare needs. The term *“new form of care"* is defined by the Innovation Fund (IF) of Germany's Federal Joint Committee as *“a structured and legally binding cooperation between different professional groups and/or institutions in medical and non-medical care”*.

The nFC-isPO is part of the isPO project funded by the IF. It is implemented in four local cancer centres and is currently undergoing a continuous quality improvement process. As part of the isPO project the nFC-isPO is being evaluated by an independent institution: the Institute for Medical Sociology, Health Services Research, and Rehabilitation Science (IMVR), University of Cologne, Germany.

The four-year isPO project was selected by the IF to be eligible for funding because it meets the requirements of the federal government's National Cancer Plan (NCP), in particular, the *“further development of the oncological care structures and quality assurance"* in the psycho-oncological domain. An independent evaluation is required by the IF to verify if the new form of care leads to an improvement in cross-sectoral care and to explore its potential for permanent integration into the German health care system.

**Methods:**

The nFC-isPO consists of six components: a concept of care (C1), care pathways (C2), a psycho-oncological care network (C3), a care process organization plan (C4), an IT-supported documentation and assistance system (C5) and a quality management system (C6). The two components concept of care (C1) and care pathways (C2) represent the isPO clinical care program, according to which the individual cancer patients are offered psycho-oncological services within a period of 12 months after program enrolment following the diagnosis of cancer. The remaining components (C3-C6) represent the formal-administrative aspects of the nFC-isPO that are intended to meet the legally binding requirements of patient care in the German health care system. With the aim of systematic development of the nFC-isPO while at the same time enabling the external evaluators to examine its quality, effectiveness and efficiency under conditions of routine care, the project partners took into consideration approaches from translational psycho-oncology, practice-based health care research and program theory. In order to develop a structured, population-based isPO care program, reference was made to a specific program theory, to the stepped-care approach, and also to evidence-based guideline recommendations.

**Results:**

The basic version, nFC-isPO, was created over the first year after the start of the isPO project in October 2017, and has since been subject to a continuous quality improvement process. In 2019, the nFC-isPO was implemented at four local psycho-oncological care networks in the federal state North Rhine-Westphalia, in Germany. The legal basis of the implementation is a contract for *"special care"* with the German statutory health insurance funds according to state law (§ 140a SCB V; Social Code Book V for the statutory health insurance funds). Besides the accompanying external evaluation by the IMVR, the nFC-isPO is subjected to quarterly internal and cross-network quality assurance and improvement measures (internal evaluation) in order to ensure continuous quality improvement process. These quality management measures are developed and tested in the isPO project and are to be retained in order to ensure the sustainability of the quality of nFC-isPO for later dissemination into the German health care system.

**Discussion:**

Demands on quality, effectiveness and cost-effectiveness of in the German health care system are increasing, whereas financial resources are declining, especially for psychosocial services. At the same time, knowledge about evidence-based screening, assessment and intervention in cancer patients and about the provision of psychosocial oncological services is growing continuously. Due to the legal framework of the statutory health insurance in Germany, it has taken years to put sound psycho-oncological findings from research into practice. Ensuring the adequate and sustainable financing of a needs-oriented, psycho-oncological care approach for all newly diagnosed cancer patients, as required by the NCP, may still require many additional years. The aim of the isPO project is to develop a new form of psycho-oncological care for the individual and the population suffering from cancer, and to provide those responsible for German health policy with a sound basis for decision-making on the timely dissemination of psycho-oncological services in the German health care system.

**Trial registration:**

The study was pre-registered at the German Clinical Trials Register (https://www.drks.de/DRKS00015326) under the following trial registration number: DRKS00015326; Date of registration: October 30, 2018.

## Background

The annual incidence of new cancer cases is increasing significantly worldwide [1]. In Germany almost 500,000 new cases of cancer were diagnosed in 2016, and this number is expected to increase by at least 23% between 2010 and 2030 [2]. The number of cancer survivors will also grow steadily, largely due to the ageing of the baby boomer generation [1–3], but also due to targeted cancer therapies that are increasingly tailored to the individual patient [4, 5]. Cancer patients are regularly affected by high emotional distress, and consequently need informational and psychosocial health care at various stages of their cancer continuum [6–9]. Distress and unmet supportive care needs seem to be affiliated with each other [10–12]. Emotional distress is at its highest in the acute phase of cancer therapy, and in most cases decreases continuously over the following months and years [13–15]. Up to 35% of cancer patients suffer from mental disorders such as anxiety, depression or adjustment disorders [16–18]) and up to 50% suffer from emotional distress made up of anxiety, depression, or both [6, 19, 20]. The presence of anxiety and depression in cancer patients is associated with greater healthcare utilization [21–23]. In general, the risk of emotional distress is considered significantly higher in cancer patients than in the general population [19, 24]. In recent years, psycho-oncological intervention research has developed evidence-based interventions that are sufficiently effective in alleviating the psychosocial problems, emotional distress, impaired quality of life, anxiety, depression and other mental impairments in cancer patients [25–27]. The health economic benefit of psycho-oncological care is also becoming increasingly apparent [28, 29].

The growing body of evidence-based knowledge on the emotional and psychosocial health care needs of cancer patients and effective psycho-oncological interventions to alleviate them, has led to increasing demands aimed at health policy makers to transfer this knowledge into patient care [30–33]. This is accompanied by a growing emphasis on the psycho-oncological support of cancer patients along their individual cancer care continuum [30, 34].

In Germany, the National Cancer Plan (NCP) calls for the integration of the psycho-oncology domain into bio-medical cancer therapy and cancer aftercare [35]. At least since 2010, psycho-oncology has endeavoured to transfer scientific findings into clinical practice and to each individual patient [36–39]. The overarching framework for bridging the gap from bench to bedside is provided by translational psycho-oncology and considerations on the development, implementation and evaluation of psycho-oncological care services [39–41]. Internationally, the development of practice guidelines began around 2008 and represents an important milestone in the implementation of psycho-oncological research into practice [31, 42, 43]. In Germany the first evidence-based guideline on psycho-oncological diagnosis, counselling and treatment of adult cancer patients was published 2014 by the AWMF (Arbeitsgemeinschaft der Wissenschaftlichen Medizinischen Fachgesellschaften), a network of the scientific medical societies in Germany [44]. The AWMF guideline obliges each institution to develop and implement a written concept for psycho-oncological patient care in terms of a quality feature [44]. Despite considerable efforts in the development, implementation and dissemination of psycho-oncological services to enable guideline-based care for the target population, significant progress still needs to be achieved, both internationally [32, 38, 45] and in Germany [46–48]. The attempts to adapt the published guidelines for screening, diagnostics, intervention and evaluation to the conditions of local institutions (local tailoring), for the coordination and management of patient care, for inter-professional cooperation, or for documentation and quality assurance (local tailoring), reveals the challenges faced by evidence-based clinical practice [48–54]. Consequently, only about 9% of 6,000 cancer patients interviewed in Germany reported that they received psycho-oncological care during their hospital stay, and only 3% took advantage of a psychosocial cancer consultation centre [55]. In contrast, the empirically justifiable need for psycho-oncological care concerns around 50% of all cancer patients [6] and approximately 40% of cancer patients request these services [56]. According to a recent German study, psycho-oncological care in cancer centres or rehabilitation facilities is provided for up to 28% of patients [57]. Thus, the goal of the National Cancer Plan that *"all cancer patients receive adequate psycho-oncological care as needed"* [35] is still far from being achieved.

## Methods

### Tasks and objectives of the isPO project

The health care system of Germany is regarded as a highly complex self-governing system [58, 59]. The Innovation Fund (IF) was established 2015 at the Federal Joint Committee by the Federal Ministry of Health, the highest decision-making body of the German healthcare system [60]. The IF aims to improve the complex health care system of Germany and the health care of the German population. Since 2016, the IF has provided several million euros of support to innovative, particularly new, cross-sectoral forms of care. “*New forms of care”* (nFC) are forms of healthcare that not only establish a structured and legally binding cooperation between different professional groups and institutions, but also go beyond the current, sometimes fragmented, in-patient and out-patient healthcare provided by the statutory health insurance in Germany. Funding is given to care models that improve cross-sectoral care for the federally insured population, optimize intersectoral interfaces, or entail concepts to overcome the separation of in-patient and out-patient health care sectors.

In 2016, the **C**entre for **I**ntegrated **O**ncology at the University Hospital Cologne (CIO-Cologne), together with consortium partners at the universities of Cologne and Dortmund, as well as the Cancer Society NRW e.V., the House of Cancer-Patient Support Associations of Germany, Federal Association e.V. and three large statutory health insurance companies in Germany (BARMER, Techniker Krankenkasse and AOK Rheinland/Hamburg) have applied successfully to the IF with the project, integrated, cross-sectoral psycho-oncology – isPO [61]. The isPO project contributes to the implementation of the German National Cancer Plan (NCP), which calls for the further development of *"oncological care structures and quality assurance”* (field of action 2 of the NCP [35, 61]). Within field of action 2, action goal 9 of the NCP requires that *"all cancer patients receive appropriate psycho-oncological care when needed"*. This is to be achieved by better recognition of the specific needs for psychosocial support and mental disorders of cancer patients that require treatment, and by ensuring the necessary psycho-oncological care in the in-patient and out-patient settings. These NCP goals need to be successively implemented in the German health care system with reference to the national guideline on psycho-oncology [44]. The implementation of these recommendations is monitored within the framework of the certification of oncological centres in Germany [62].

### isPO project duration and phases

The isPO project is scheduled to run from October 2017 to September 2021. It is carried out in four consecutive phases [63].

#### Phase 1 (10/'17–09/'18)

**Fig. 1:**
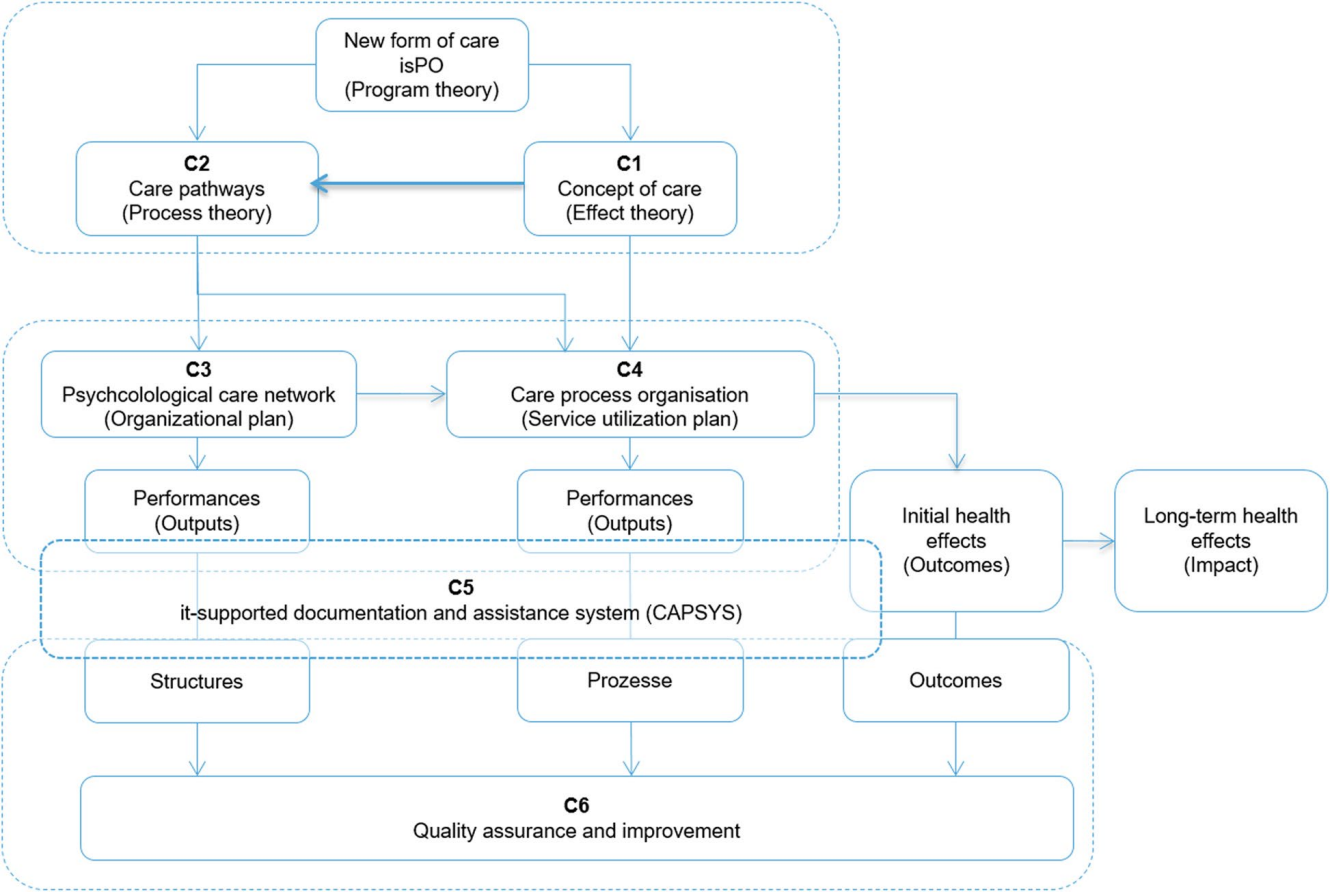
Components and features of the new form of care isPO. (references to the program theory according to Issel [66] are shown in brackets)

Development of the nFC-isPO and the isPO care program (see Figs. 1 and 4; Table 1); preparation of the contract for *"special care"* according to § 140a SCB V [58] in which eight major statutory health insurance companies in Germany are involved; establishment of psycho-oncological care networks at four local care locations in the Rhineland North Rhine-Westphalia (Centre for Integrated Oncology, University Hospital Cologne; Johanna Etienne Hospital, Neuss; Maria Hilf Clincs, Mönchengladbach; GFO-Clinics Troisdorf, Business premises St. Josef Troisdorf), and external prospective evaluation by the IMVR.

**Table 1:** Description of the core services and core processes of the isPO care program

**Domain of services (care area)**	**Care step/ allocation criteria**^a^	**Calculated number of services within 12 months**	**Core service functions/** **criteria**	**Description of the core service, responsibilities and performances**
**Overall objective of the isPO care program**				Empowerment of patients to actively participate in their cancer therapy and aftercare while maintaining the highest possible quality of life
**Case-management**	step 0 / all enrolled patients	up to 20	coordination	Coordination and organization (care and case management: scheduling, correspondence, meetings, reporting, quality assurance, service accounting etc. at patient and organizational level) Contact person for disease-related and/or treatment-related psychosocial questions in the cancer continuum, especially for patients who are not treated at levels 2 and 3
			intake	Ensuring the first contact with the patient shortly after the medical referral, in-depth information and enrolment of the patient
			assessment	Collection of all necessary information (among other things implementation of the screening) at intake
			planning/linking	Documentation of test results in CAPSYS-docu and assignment of the patient to the care providers according to the automated criteria-based data evaluation within CAPSYS-docu (see Fig. 4 and 5)
			monitoring	Monitoring of the path-guided course of care using CAPSYS-assist, information from the treating physicians as well as the patients on the screening results by means of automatically generated CAPSYS-reports, documentation for service billing, contact person for patients and the service provider team on organizational questions
			re-/evaluation	Data collection and documentation in CAPSYS-docu on T2 (4th month of treatment) and T3 (12th month, end of treatment); information from the treating physicians as well as the patients on the screening results by means of automatically generated CAPSYS reports
**isPO-oncoguide**	step 1 / all enrolled patients	up to two	orient	Provision of information and explanations to promote patient self-help resources by a trained volunteering person previously ill with cancer
			inform	Information on community-based psychosocial support services, contacts to non-profit self-help groups, services offered by statutory health insurance companies and internet addresses of non-profit cancer societies and other independent and evidence-based sources of information and support, including the delivery of written information material to the patient
			explain	Provision of patient information on the basis of a self-commitment declaration signed by the trained person (in particular: empathetic and sympathetic attitude, listening to the patient, no medical or therapy-related or legal advice, restraint should the patient request personal advice, not holding their own medical history as a standard, complying with the head of the psycho-oncological care unit)
			agree	Documentation on whether the patient agrees to having experienced the information received as helpful
			document	Assessment of the personally felt quality of the information meeting with indication of a possible further need for care on the part of the patient (note: the documentation in CAPSYS-docu is done by a case manager)
**Psychosocial care**	step 2 / HADS-G ≥ 15/ PSR ≤ 3	up to six	orient	Accompaniment of the patient during their cancer disease and cancer therapy with the aim of improving their individual skills in coping with symptoms and treatment, as well as their short and longer-term physical and psychosocial consequences, including the life changes necessary to live with the protracted disease
			assessment	Assessment of the severity of the patient’s psychosocial problems, based on the results of a questionnaire and a half-structured interview and creation of a psychosocial self-help plan
			advice	Consultation on necessary care needs based on the psychosocial self-help plan and preparation of a priority list of psychosocial care needs
			agree	Joint prioritization of the psychosocial issues that need to be dealt with first
			assist	Determination of the professional support needs of a patient based on the assessed severity of the problem Low need of support: the patient receives information or advice on how to independently carry out the interventions defined in the help plan High need of support: The patient is actively supported and guided by the psychosocial expert or the service provider implements aspects of the help plan for instead of with the patient
			arrange	Accompanying support of the patient in the implementation of their self-help goals (e.g. internet research, discussions with treating physicians or other persons, provision of documents for applications to authorities)
**Psycho-oncological care**	step 3a / HADS-G ≥ 15	up to 14	orient	Accompaniment of the patient during their cancer disease and cancer therapy with the aim of the following: reduction of psychological burden; emotional stabilization; improvement of self-management in coping with acute physical symptoms, treatment related or acute states of emotional dysregulation; coping with the short and longer-term physical and psychological consequences of cancer, including the life changes necessary for living with a protracted disease
			diagnose	Assessment and classification of the severity of the patient's psychological burdens based on the results of the screening assessment at intake and an initial psycho-oncological examination. In particular, specification of the nature and severity of the emotional distress or mental disorder on the basis of available information
			indicate	Selection of a suitable psychotherapeutic intervention for the current problem of a patient. In the continuum of a bio-medical cancer therapy, psychotherapeutic diagnostic and therapeutic decisions may repeatedly become necessary. In the care area on step 3a/3b CAPSYS-assist offers decision support for finding a possible indication based on questionnaire data, anamnesis data, protocol data etc
			intervene	Selection and implementation of one of 25 treatment modules described in the isPO manual for the care areas at step 3a/b
			evaluate	Continuous evaluation of the achievement of psychotherapeutic goals
**Complex care**	step 3b / HADS-G ≥ 15/ PSR ≥ 6	up to 14 as in step 3a and up to 4 as in step 2	orientate	Accompaniment of the patient during their cancer disease and cancer therapy as in 3a Psycho-oncological treatment as in care area of step 3a; psychosocial care as in care area of step 2, following a regulation by the psychotherapist

^a^The allocation criteria are described in the paragraph: p*atient allocation and follow up within the isPO care program*

Abbreviations: *HADS *Hospital Anxiety and Depression Scale (HADS) [64] *PSR* Psychosocial Risk questionnaire (German: Psychosozialer Risikofragebogen) [65]

#### Phase 2 (10/'18–09/'19)

Implementation of the nFC-isPO into the local psycho-oncological care networks (*N* = 1.610 patients; first patient in, January 2019), accompanied by quarterly network-internal quality circles; quarterly network-wide participatory quality development [67] workshops with participation of the service providers of the local networks, the consortium partners in the isPO project and the participating statutory health insurance companies; first phase of the external formative evaluation by the IMVR.

#### Phase 3 (10/'19–09/'20)

Continuation of patient care within the nFC-isPO framework (last patient in, September 2020); local and network-wide quality improvement; second phase of the external formative evaluation by the IMVR.

#### Phase 4 (10/'20—09/'21)

End of patient care within the framework of nFC-isPO in the local networks (last patient out, September 2021); external summative evaluation by the IMVR.

The isPO project entails the “*new form of care*”, integrating the isPO care program, and the isPO study. The isPO study is conducted by the external, project-independent Institute for Medical Sociology, Health Services Research and Rehabilitation Science (IMVR) at the University of Cologne, Germany [68]. The purpose of the evaluation is to assess the quality, the effectiveness and the cost-consequences of the isPO care program [69]. The consortium partners are responsible for special aspects of the development, implementation, and evaluation of the Components of the nFC-isPO. The health insurance funds are supporting the isPO project partners to ensure that the nFC-isPO is carried out in compliance with the German Social Code Book 5 (SCB V) of the statutory health insurance system. The isPO program should be implemented in the local health care facilities on the basis of a contract for *"special care*" according to § 140a SCB V. Therefore, the nFC-isPO is tailored for the implementation into the German health care sector. Contracts for *"special care"* according to SCB V stipulate that, in addition to clinical services of patient care (the isPO care program), formal-administrative services are also provided, which, in particular, serve patient enrolment, service coordination between different professional groups, contract-compliant management of patient care, and quality assurance and billing.

A specific project plan had to be introduced in order to develop and implement isPO and evaluate the isPO project within the given framework conditions of the sponsor (Innovation Fund at the Federal Joint Committee), the German NCP, the national guideline psycho-oncology and the German Statutory Health Insurance System.

### The isPO Project plan

The isPO project is based on a two-part project plan:

(1) a plan for the development, implementation and internal evaluation of the new form of care (as shown below), and.

(2) a plan for the independent external evaluation of the isPO care program [69].

While the isPO project is funded, both plans are combined, linking quality assurance and external evaluation within this time period.

During the project development phase (October 2017 to December 2019), the nFC-isPO has been developed against the background of Issel´s program theory ([66], see chapter: *components of the new form of care isPO*). The development process was externally explored with prospective evaluation [69]. During the implementation phase (since January 2019—ongoing), the internal evaluation entails quarterly cross-network participatory quality development workshops with subsequent evaluations to continuously improve the application of the nFC-isPO. This process is accompanied by the external evaluation via measures of two formative evaluation phases [69]. The evaluation phase of the isPO project (starting in October 2020) will include an external summative and outcome evaluation [69].

### Scientific background of the development and implementation of the nFC-isPO

In their *“roadmap to excellence”*, renowned psycho-oncologists advocate a constant mantra ([70] p. 572) *“the biopsychosocial perspective is the foundation for maximizing the benefit of medical care and to promote quality of life. This can only be achieved with psychosocial services that are at least as good as the medical care provided”*. Consequently, psycho-oncological forms of care whose clinical and service quality will come close to that of medicine must be developed, implemented and refined with a scientific background that comes close to the service quality of medical cancer care.

The nFC-isPO is based on a scientific concept [71] consisting of translational research [40, 72], practice-based health care research [73, 74] and the program theory according to Issel [66]. The development of the isPO care program itself was based on a population and need-based stepped care, the emotion regulation approach of *"clinical psycho-oncology"* according to Kusch, Labouvie and Hein-Nau [75] and a clinical pathway that was successively implemented at the **C**enter for **I**ntegrated **O**ncology Cologne – Bonn, at the University Clinic of Cologne (CIO-Cologne [76]). A major goal of the isPO project is the transfer of this *"local practice model"* of the CIO-Cologne into a care program that guarantees a comparable clinical quality of patient care for the population suffering from cancer and across local oncological centres providing different hospital care levels and geographical coverage.

In the German health care system, all services that are financed and provided within the framework of SCB V must not only comply with the current state of the art in the respective field with but also with more formal-administrative requirements. The services must be sufficient, appropriate and economical; they must not exceed what is necessary (§ 12, para. 1, SCB V; Economic efficiency dictates). The NCP [35] and the national guideline on psycho-oncology of the AWMF [44] also make clinical and formal-administrative demands on the field of psycho-oncology. To meet these requirements, the isPO care program includes considerations of emotion regulation according to Gross [77–82], psycho-oncological guidelines [44, 83–85] stepped care models of psycho-oncology [86–88] and the legal requirements of the SCB V. Moreover, attention to these aspects enables the external evaluation institute to record, analyse and evaluate the clinical and formal-administrative quality of the nFC-isPO and the isPO care program [69].

*Translational psycho-oncology:* In oncology, the term *"translational"* refers to application-oriented cancer research, or to the effort of bridging the gap between *"bench and bedside"*, in which basic science (**T**ranslation **0**) is transferred to research on humans (**T1**) and then to the bedside, with the use of clinical studies and other research findings (**T2**) [40, 89, 90]. The question of how evidence-based knowledge can be transferred from *"bedside-to-practice"*, for example from clinical research in university hospitals (**T2**) to clinical practice in community hospitals (**T3**) is the concern of practice based research [72] or clinical outcome research [91]. Implementation and dissemination research is concerned with knowledge translation from clinical outcomes and practice-based research *"to-community"*, that is concerning the entire population within a national health care sector (**T4**, [91, 92]).

The isPO project is, therefore, less about gaining new knowledge (see however [69]) than about the translation of scientifically sound knowledge into clinical practice at the level of university and community hospitals. In particular it is about the quality and safety of patient care, i.e. the question of whether and how psycho-oncology can offer the *“right care”* to the *“right patient”* at the *“right time”* in the *“right place”* [71, 93–95]. For the concept of "*right*" in patient care, see Olsen et al. [96–98].

*Practice-based health care research:* According to Westfall et al. ([72], p 404) *“practice-based research occurs in the office, where most patients receive most of their care most of the time and may be the essential link between bench discoveries, bedside efficacy, and everyday clinical effectiveness. Practice-based research and practice-based research-networks (PBRNs) may help because they can:*

1. *identify the problems that arise in daily practice that create the gap between recommended care and actual care;*
2. *demonstrate whether treatments with proven efficacy are truly effective and sustainable when provided in the real world setting of ambulatory care; and*
3. *provide the “laboratory” for testing system improvements in primary care to maximize the number of patients who benefit from medical discovery.”*

This conception of the practice-based research approach is a perfect fit for the isPO project.

Within the isPO project the development, implementation, and evaluation of the nFC-isPO and the isPO care program is based on consecutive practice-based research phases [71, 73, 74]:

- *Concept development*, in particular the development and presentation of the psycho-oncological care concept and the care pathways through which the patient care services that have been founded on scientific evidence to be *“right”* are expressed.
- *Accompanying formative evaluation* in which the provision of patient care is evaluated with regard to predefined recommendations.
- *Quality improvement*, which uses the findings of accompanying research results directly to improve the care provided to patients.
- *Outcomes research* that seeks to answer the questions of the appropriateness of patient care under the aspects of quality, effectiveness and cost-consequences.

*Program theory:* The program theory according to Issel [66] enables the structured development of specific components of a new form of care (according to the concept development phase of practice-based research; see chapter: *components of the new form of care isPO*).

The structured development process makes the components accessible for the accompanying formative evaluation, which in the case of isPO is conducted by the independent institute IMVR, and the continuous improvement process, which is pursued through network-internal quality assurance and cross-network quality improvement measures.

## The components of the new form of care isPO

The new form of care isPO (nFC-isPO) was developed based on the program theory according to Issel [66], which was adapted in parts to meet the requirements of the isPO project (compare Fig. 1).

Issel’s program theory [66] provides a regulatory framework within which external requirements of health care research and health care legislation and internal organizational, technical or practical necessities of a health care organization can be transferred into a coherent form of care. In the case of isPO, the external requirements come from the IF, the NCP, national guidelines for psycho-oncology, the state of knowledge in psycho-oncology, and the legal framework of the Social Code Book V, on which the contract for *"special care"* according to § 140a SCB V is based.

The institution-internal, formal-administrative necessities resulted from the organizational structure of each participating hospital, the technical and practical processes of in-patient and out-patient cancer care, and general aspects such as documentation obligations, data protection, quality assurance, or working time regulations and accounting. All of these external and internal aspects were taken into account in the development of the nFC-isPO.

### Component 1 (C1) – care concept

The national evidence-based guidelines on *“psycho-oncological diagnosis, counselling and treatment of adult cancer patients”* [44] requires each health care institution involved in the treatment of cancer patients to develop and implement a written concept for psycho-oncological patient care, and this should be demonstrated as a quality feature. The concept should consider the guidelines’ recommendations and include core statements about the contents, goals and working methods of the psycho-oncological service provision. The concept should be in line with the state of scientific knowledge in psycho-oncology and be updated regularly.

The development of the care concept within the nFC-isPO is based on the *“effect theory”* (C1) according to Issel ([66], see Fig. 3). The state of scientific knowledge in psycho-oncology and psychosocial services represents an extension of the *"clinical psycho-oncology"* approach according to Kusch, Labouvie and Hein-Nau [75, 99]. For the purposes of specific patient care the contents, goals and working methods of psycho-oncological service are transferred from the care concept into care manuals. The care concept of nFC-isPO is presented in the chapter: c*are concept of the isPO care program*.

### Component 2 (C2) – care pathways

The care pathways serve to transform the guideline recommendations into concrete performance specifications for the provision of patient care [31, 41, 83, 99, 100]. Within the care pathway of the nFC-isPO, the core elements of the isPO care manuals are presented in the form of pathway algorithms [101] and operational defined selection and execution recommendations [74] that have been linked to the pathways in order to make concrete recommendations for action available to service providers (see Fig. 3 and chapter: C4—*care process organization*).

The development of care pathways in the nFC-isPO is based on the *“process theory”* (C2) according to Issel [66] and the formal structure of the recommendations is based on the concept of *“care psychology”* according to Kusch [74]. The basic care pathway of nFC-isPO is presented in the paragraph: *care pathways of the isPO care program*. Within the nFC-isPO, the care concept and the care pathways represent the isPO care program.

### Component 3 (C3) – psycho-oncological care network

The NCP calls for psycho-oncological care to be provided in the in-patient and out-patient health care sectors, and the IF demands that new form of care should ideally overcome the separation of in-patient and out-patient health care sectors. Within the nFC-isPO the isPO care program is to be integrated into bio-medical care and is to cover the acute cancer therapy and cancer aftercare [61]. For the reasons listed above, the organizational plan of the nFC-isPO consists of a network of both cancer centres and out-patient care providers in independent practice, so that cancer patients can receive psycho-oncological care regardless of whether they are in an hospital, or in a primary medical care facility, or temporarily receive no medical therapy at all. The cooperation of these organizations and the coordination of patient care within and between these organizations requires a contractual basis.

Within the psycho-oncological care network the contracts should regulate both the vertical care management (hospital and primary care management, doctor's office, oncological departments, psycho-oncological division, quality management unit, accounting office) and the horizontal case management (doctors and nurses responsible for cancer therapy, case managers, psychosocial and psychotherapeutic service providers). In the isPO project, the contract for *"special care"* according to § 140a SCB V formed the external basis of the organizational structure of the psycho-oncological care network and care process organization. Agreements on the intra-organizational care and case management, on the implementation of the quality requirements from the special care contract, the service accounting, documentation and data protection have been laid down in an *“isPO cooperation agreement”* between each cooperating psycho-oncological care network and the consortial leader organization at the University Clinic of Cologne. And finally, the agreements on the internal organization and cooperation among the psycho-oncological care network institutions and professional groups involved in the development and implementation of the nFC-isPO and the isPO care program were outlined in an organization chart. This organogram serves to illustrate the cooperation structures of the network and to assign specific tasks and powers in the care process organization (care and case management) to the individual professions.

### Component 4 (C4) – care process organization

Cancer care centres should ensure the delivery of high-quality psycho-oncology care and integrate the recommended health care delivery in real-world settings of the cooperating institutions and professions [32, 38, 91]. The legal basis on which the insured person receives care within the psycho-oncological care network is specified in the special care contract with health insurance agencies. This special care contract refers not to all services, but rather to the core clinical services and core clinical processes of the care concept and the care pathways. But above all, it specifies the core formal and administrative services to be provided by the contracting party.

**Fig. 2:**
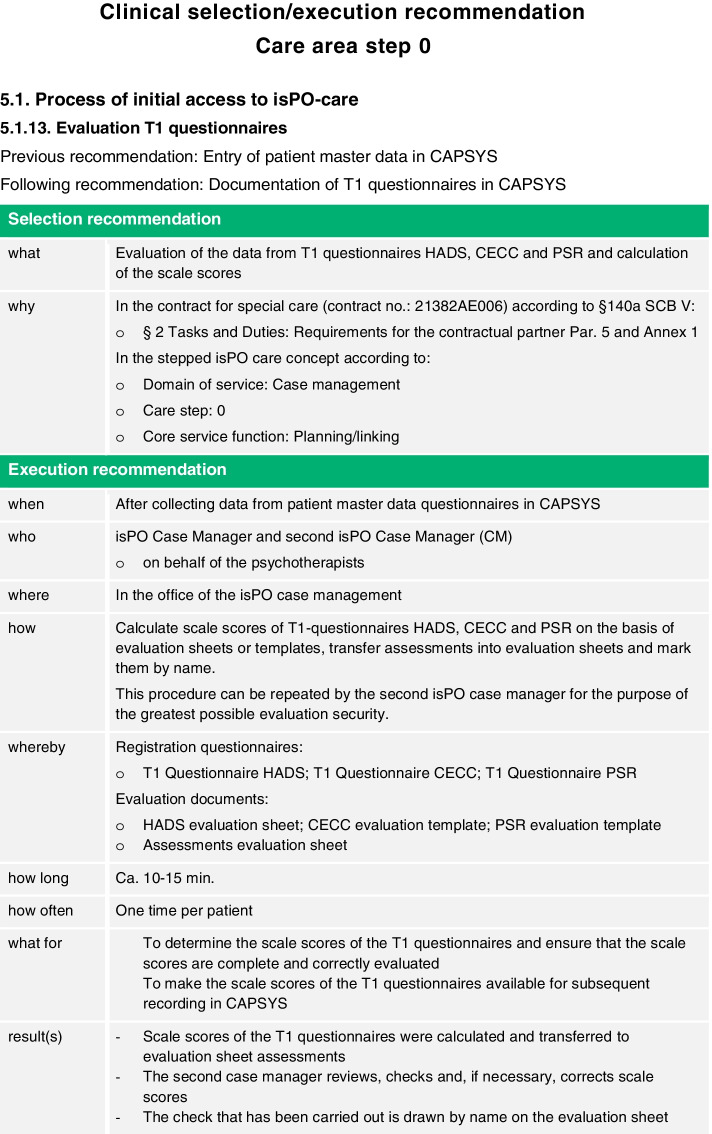
Clinical selection/execution recommendations for care area step 0: 5.1 Process of initial access to isPO-care

In order to provide the clinical as well as the formal-administrative services of the nFC-isPO in a contractually defined form, the core services (*"doing the right thing"),* and the core processes (*"doing the right thing right"*) were operationalized in the form of selection and execution recommendations according to the concept of “*care psychology*” ([74] see Fig. 2 as example).

- *Selection recommendations* substantiate why something is the right thing to do in clinical patient care or in administrative care management and specify the respective evidence or legal basis (*"doing the right thing"*).
- *Execution recommendations* specify the “*w-elements*” of service provision; what, how (*“wie”* in german language), which materials, by whom, and with which results a specific core service should be done (*“doing the right thing right”*).

More than one hundred recommendations are integrated into the care pathways algorithms of the isPO program. These pathways represent both the *“state of the art”* of the care concept and the *“right form”* of its implementation in real patient care settings.

The operationalized recommendations specify both the clinical and formal-administrative quality of the provision of the services as stipulated in the care concept and the special care contract^1^ with the health insurance agencies. Thus they, in turn, form the basis for the specification of all other components (C3-C6) of the nFC-isPO.

The first measures in the specification of the care process organization plan were to:

- draw up the organization chart of the psycho-oncology network,
- determine the persons responsible for the psycho-oncological network organization,
- find those responsible for the clinical and formal-administrative domain of the care program (leading isPO-team members), and
- assign the roles and responsibilities of the executive isPO team members according to the recommendations.

The roles and responsibilities of the management and operative isPO team members serve to ensure an appropriate implementation of the nFC-isPO under the specific hospital and socio-geographical conditions of the local psycho-oncological care network. However, since the clinical and formal-administrative recommendations cannot dictate the concrete, routine performance of a local network, the recommendations are subject to local adaptation and tailoring by the employees of each local care network. Local tailoring of predefined recommendations is an important element in ensuring that recommended care services are actually implemented appropriately in a local network, are accepted by the service providers, and can therefore reach every patient [102–104]. If tailoring to the local health care institution is neglected, performance often fails to meet the requirements of the end-users (patients), the service providers (isPO team members), and the external stakeholders (health insurance companies, health policy, society). Therefore, most of the clinical selection and implementation recommendations are explicitly not to be understood as directives or regulations, but as guidelines in the sense of a *“support for action”*.

The implementation of these clinical recommendations is therefore not further specified in a more specific written form, but rather is supervised by the clinical management of the local care network and within the framework of the local quality assurance measures. On the other hand, the formal-administrative recommendations resulting from the special care contract are to be understood in the sense of *"standard operating procedures"* (SOP). These SOP´s relate to the basic allocation criteria for implementing the care pathway (see Fig. 5), patient enrolment, documentation and information requirements, regular reports to the health insurance funds, or internal quality assurance measures. A local isPO network coordinator monitors the appropriate implementation of the SOP´.

Both the selection and the execution recommendations are the central components of both the isPO quality assurance and improvement measures (C6), and the isPO specific IT-system (C5), in which they are at hand accessible to the service provider.

### Component 5 (C5) – information technology (IT)-supported documentation and assistance system CAPSYS (“Computerbasiertes Assistenzsystem Psychoonkologie”, computer-based assistance system psycho-oncology).

Since the introduction in 2016 of the "Law for Secure Digital Communication and Applications in the Healthcare System" (E-Health Law), the German healthcare system has increasingly pursued the goal of secure digital communication between doctors, hospitals, health insurance companies, and patients [105]. In the field of oncology, digitization should also contribute to a *"knowledge-generating care"* and, among other things, efficiently connect interfaces between existing hospital information systems and databases of decentralized network partners [106]. With the IT-supported documentation and assistance system CAPSYS, the isPO project aims to introduce and test digitalization in the field of psycho-oncological care.

CAPSYS was developed to meet the many external requirements of the special care contract according to SCB V and the internal needs of service documentation, care and case management, quality management, billing or data protection as specified in the *“isPO cooperation agreement”*. All these tasks are performed in the German health care system by different departments, different documentation systems and different documentaries. The isPO project is testing whether these tasks can be fulfilled by one computer system alone.

CAPSYS consists of two parts:

1. CAPSYS-docu, for recording core data of patient care and contractual service provision, and
2. CAPSYS-assist for planning, guidance and examination of a guideline-supported and quality-assured patient care.

Both parts are interlinked in a special way. In CAPSYS-docu the specific services of clinical patient care and the fulfilment of formal-administrative responsibilities are recorded in specific documentation modules, and also exclusively by the predefined service providers treating patients at specific care areas (see paragraph: *stepped-care approach of the intervention theory*). The individual documentation modules are structured so that they enable the recording of the services provided in each care area and for each care function of the isPO care program (see Table 1). In addition, the individual documentation modules are linked to each other so that they correspond to the process of service provision as predefined in the care pathways. From the respective documentation modules, the service provider (e.g. psychotherapist, psychosocial worker) can directly access via CAPSYS-assist the pathway algorithm and the operational recommendations for their area of care. Consequently, they can orient themself directly in the selection and execution recommendations, allowing for the recommended quality of the service provision tailored to the actual phase and area of patient care in each individual case. Thus, the data recording in CAPSYS-docu also ensures that the legally and contractually stipulated parameters are collected and that at the same time the recommended quality of patient care is reflected in the real care of a specific individual case (CAPSYS-assist). Thus, CAPSYS-docu helps to increase the likelihood that the right patient is treated within the right care area, by the right service provider, with the right qualifications, and with the right services, and, at the same time, ensures that these services are billed correctly.

Once the performance characteristics of the structures, processes and outcomes of patient care and case management within a local care network have been recorded, continuous databased quality assurance and improvement becomes possible within the care network. In the nFC-isPO this is realized by CAPSYS-assist (see *component C6—quality assurance and improvement*).

### Component 6 (C6) – quality assurance and improvement

Quality assurance is concerned with reviewing the performance of products and services during use [107]. Quality improvement aims to systematically and continuously solve problems in healthcare, improve service provision, and ultimately provide better outcomes for patients [108]. The isPO project combines both approaches.

In the local psycho-oncological care network, internal quality circles are held every quarter to ensure the quality of care. In addition, together with the consortium partners in the isPO project, external quality workshops are held every quarter for continuous quality improvement. This is done on the basis of a network quality control plan drawn up by the consortium management for the cross-network monitoring and quality improvement of the local networks involved in the isPO project.

According to Issel [66], each element of an organization plan (C3) and a service utilization plan (C4) must be accompanied by outputs and outcomes that reflect the performance of a program (see Fig. 1). In the field of psycho-oncological research, outputs correspond to the performance indicators of the structure and process quality of care [109, 110]. Outcomes are indicators of the outcome quality, that is, the initial effectiveness of patient care provided [111]. The evaluation of long-term health effects (impact) is usually not the subject of quality assurance measures within a health care institution, but a measure at the level of health policy. However, the governance and management of a psycho-oncology care network requires clinical and formal-administrative indicators to monitor the psycho-oncology care process, assess patient care performance and evaluate the added value [112] of providing the isPO care program.

In order to be able to measure the performance of a psycho-oncological care network, concrete and valid measures of the outputs (structures, processes) and outcomes are needed [113]. The term *"concrete"* refers to the possibility of measuring unambiguously defined indicators and the term *"valid"* refers to the fact that these indicators are suitable for making statements on the clinical and formal-administrative quality of a psycho-oncological care network.

Within the nFC-isPO the defined indicators are derived from the operational, clinical and formal-administrative recommendations of the care pathways. The reference of the indicators to the recommendations thus also gives them their validity and relevance for quality assurance and quality improvement measures within and across networks. The required basic data for the generation of the output and outcome indicators are collected at the time when the service provider records specific aspects of the services provided during their patient care in CAPSYS-docu.

In CAPSYS-assist the recorded data are processed and transferred to database quality reports. On the basis of these database quality reports and with the use of the suitable selection and execution recommendations, the local network coordinator carries out the quarterly internal network quality circles. Since the quality reports of different local networks are also sent to a central coordinating body (the consortial leadership within the isPO project), benchmarks of local performances can be drawn up there, and in turn be used for participatory quality improvement measures during the quarterly cross-network quality workshops. Finally, this nFC-isPO concept of local and overarching quality management allows the external evaluation organization [69] to carry out their independent prospective and formative evaluation, as required by the IF.

During the isPO project the results of the accompanying external formative evaluation support the internal process of quality improvement of the nFC-isPO and the isPO care program from an independent perspective.

## The isPO care program

**Fig. 3:**
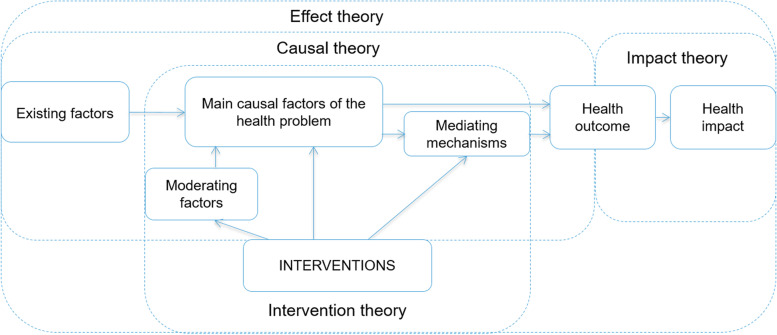
Features of the care concept of the isPO care program according to Issel [66]

The isPO care program consists of the care concept (C1) and the care pathways (C2). The care concept was designed according to the effect theory and the care pathways according to the process theory of Issel’s program theory ([66] see Fig. 3, Table 1).

### Care concept of the isPO care program

Issel’s effect theory consists of three elements: the causal theory, the intervention theory, and the impact theory [66].

The care concept of the isPO program consists of one general and four so-called minor care concepts. Each of the concepts consists of statements on the causal theory, the intervention theory and the health outcome aspects of the impact theory.

#### Causal theory

According to Issel [66], a causal theory should be specified for each care area included in a care program and these five issues should be addressed:

1. the main causal factors of a health problem,
2. the health effects associated with the health problem
3. the existing external factors affecting or linked to the problem,
4. the mechanisms by which the impact of the problem can be influenced, and
5. the health effects resulting with and without a specific intervention.

By means of the causal theory, the isPO consortial partners built a scientifically based care model that specifies constellations of the cancer patient's emotional and psychosocial burden that have to be addressed within the specific care areas targeted by the isPO care program (*“doing the right thing”*). These emotional and psychosocial burdens are described against the background of the theory of *“emotional regulation”* according to Gross [75, 77] and corresponding psycho-oncological studies [79, 80, 114–116].

**Fig. 4:**
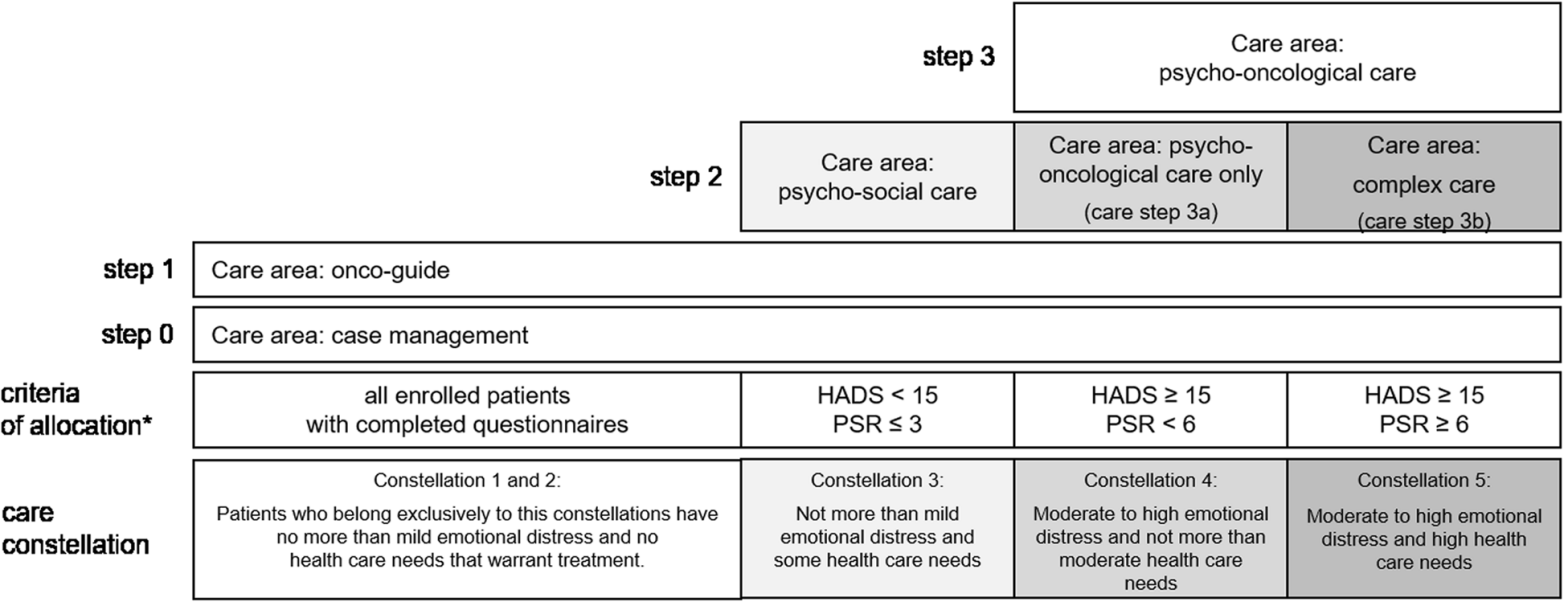
Features of the isPO care program. *The allocation criteria are described in chapter: patient allocation and follow up within the isPO care program. (HADS, Hospital Anxiety and Depression Scale (HADS) [64]; PSR, Psychosocial Risk Questionnaire (German: Psychosozialer Risikofragebogen; PSR) [65])

According to the isPO general care concept, the psychosocial and emotional burden of cancer patients can be divided into the following five care constellations ([75], see Fig. 4):

Constellation 1: Patients in need of a trustworthy organization to which they can turn with their disease-related and/or treatment-related psychosocial questions over the course of their cancer therapy and aftercare. This group consists of all cancer patients, regardless of their emotional and psychosocial burden.

Constellation 2: Patients, emotionally dysregulated because they have no sound information about their disease and the possible self-help options at hand. This group also consists of all cancer patients, regardless of their emotional and psychosocial burden.

Constellation 3: Patients in states of emotional dysregulation who do not meet the criteria of clinically relevant emotional distress, but in whom these states were caused by or associated with their disease-related and/or treatment-related psychosocial problems. These are patients experiencing no more than mild emotional distress and only some psychosocial health care needs, as determined using psychometric questionnaires (see chapter: *patient allocation and follow up within the isPO care program*).

Constellation 4: Patients suffering from specific states of emotional dysregulation such as anxiety or depression, regardless of whether these states are directly related to their cancer disease and cancer therapy or exist independently thereof. These are patients experience moderate to high emotional distress, as determined using psychometric questionnaires (see chapter: *patient allocation and follow up within the isPO care program*).

Constellation 5: Patients suffer from both specific states of emotional dysregulation and psychosocial problems. These patients experience moderate to high emotional distress and have high psychosocial health care needs, as determined using psychometric questionnaires (see chapter: p*atient allocation and follow up within the isPO care program*).

A minor causal theory has been formulated for each of the above mentioned care constellations within the framework of the general causal theory of emotion regulation in cancer patients.

#### Intervention theory

In Issel's effect theory the intervention theory is very closely interwoven with the causal theory [66]. The intervention theory defines, as far as possible, which forms and measures of an intervention address which causes, moderating factors and mediating mechanisms in order to alleviate an existing constellation of emotional and psychosocial burdens [117–124]. Within nFC-isPO, a minor causal theory and a corresponding intervention theory were formulated for each of these five care constellations. These intervention theories include theories of case management [30, 125–128] with regard to constellation 1, personalized patient information [30, 117, 129, 130] with regard to constellation 2; self-management according to the "five A's model" [131–134] with regard to constellation 3 and 5, and emotional regulation according to Gross [77, 79, 80, 116, 135] with regard to constellation 4 and 5.

#### Stepped-care approach of the intervention theory

**Fig. 5:**
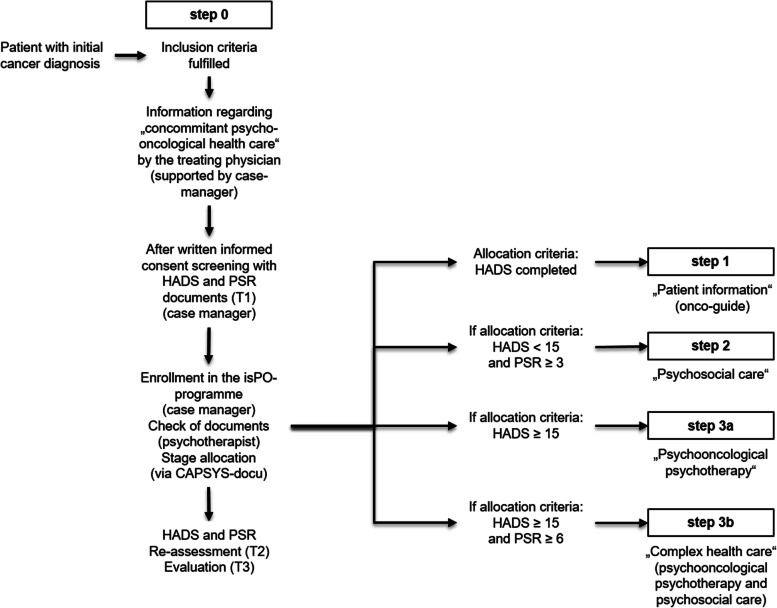
Care pathway algorithm of the contract for *“special care”* according to § 140a SCB-V of the German Social Code Book V for statutory health insurance funds. *Legend:* HADS; Hospital Anxiety and Depressions Scale [64]; PSR, Psychosocial Risk Questionnaire (German: Psychosozialer Risikofragebogen; PSR [65]); T1, assessment at entry into the isPO care program; T2 assessment within the 4^th^ month of service provision; T3 assessment at the end of psycho-oncological care (12^th^ month after program entry); allocation criteria (see chapter: p*atient allocation and follow up within the isPO care program*).

Within the isPO care concept, the intervention theory is based on a population-based stepped care approach [75, 84, 136–138], in which the available intervention measures are specifically assigned to the respective emotional regulation care constellations of a vulnerable group of patients with special health care needs. At this point we should point out that the care concept also provides a need-based stepped care approach ([76, 84, 87, 137], as each patient is allocated to a care area on the basis of predefined criteria of health care needs (see Table 1, Figs. 4 and 5).

The isPO care program consists of five care areas:

Care area at step 0: Case management (constellation 1)

Care area at step 1: isPO-onco-guide (constellation 2)

Care area at step 2: Psychosocial care (constellation 3)

Care area at step 3a:Psycho-oncological-psychotherapeutic care (constellation 4)

Care area at step 3b:Complex psycho-oncological care (consisting of care area 3a and 2 services; constellation 5)

The theories mentioned above have been chosen because they have been considered in psycho-oncological research (see the paragraph *care concept of the isPO care program*). Furthermore, these theories can also be used to derive concrete intervention measures for the creation of a structured care program (see below). While the development of the care concept determines what is considered in the care program to be the right care, the intervention theory of the care concept defines and the care manuals specify how the right care is to be properly provided (*"doing the right thing"*). Finally, the clinical recommendations for service delivery derived from the isPO care manual, together with the formal-administrative recommendations derived from the special care contract, ensure that the right psycho-oncological services are delivered to an individual patient within a specific care area (*“doing the right thing right”*).

In order to meet the requirement of providing the right care in the right way, the isPO care manuals derive the sequences of service provision within each care area from the functions and criteria of professional action of respectively professional quality of care within the corresponding intervention theories (see Table 1, Fig. 4).

Care area at step 0: The case management functions of intake, assessment, planning, linking, monitoring, re-evaluation and evaluation represent the main sequence of the psycho-oncological services ([139], see Table 1, Fig. 4).

Care area at step 1: The criteria are based on considerations regarding the provision of patient information in oncology [117].

Care area at step 2: (correspondingly also in the psychosocial part of step 3b): The functions of the self-management approaches act as a basis, as specified by the five A's model: Assessment, Advice, Agreement, Assist and Arrange [132, 140].

Care area at step 3: (and accordingly in the psychotherapy part at step 3b): The principles of therapy-oriented diagnostics in psychotherapy are applied, in particular the sequence of clinical diagnostics, indication, intervention and evaluation [141, 142].

The procedure described here enables the formulation of the above-mentioned evidence based care pathways within each care area of the stepped care approach and the operationalization of the clinical selection and execution recommendations at each step.

The selection recommendations are based on aspects of the minor causal theories of the isPO care concept and the execution recommendations on the functions and criteria of professional action derived from the minor interventions’ theories.

#### Impact theory

The impact theory specifies the short-term outcomes and the longer-term health effects that the program developers expect to emerge as outcomes and impact when implementing the health care program [66]. The short-term outcomes that the external institute IMVR is investigating in the isPO study part of the isPO project are presented elsewhere [69].

At the current stage of the isPO project (middle of implementation phase), the isPO care program does not contain any specific assumptions regarding the desired health outcomes. These will be available only at the end of the project [69].

However, as Donadebian [143] has pointed out, structures have effects on processes and these in turn influence the results. Therefore, the stakeholders financing the nFC-isPO and the managers implementing the nFC-isPO and the isPO care program according to the special care contract are very interested in insights into the links between health outputs and health outcomes. Short-term health outcomes and outputs are also a major focus in the context of quality assurance and improvement (C6), as this is ultimately about improving care structures and care processes with the aim of constantly improving patient outcomes [66].

An example of the importance of outputs for the management of local psycho-oncological care networks is the analysis of the network's internal expenditure (e.g. number of contractually agreed services/performances) for the implementation of the isPO care program. The easiest way to achieve this is to compare the calculated or presumed quantity of services (target) with the services actually provided (performance [74, 144]). In the area of quality assurance (C6) of the nFC-isPO, target performance comparisons are possible, since most of the selection and execution recommendations of the care pathways contain information on the calculated service duration (see Fig. 2, "how long") and frequency (see Fig. 2, "how often"). For example, the number of services within the 12-month duration of the isPO care program is calculated for each care area and is also specified in the care contract at detailing care steps 1 to 3b (see Table 1; *calculated number of services within 12 months*). By simply comparing the calculated (target) services with the services actually provided (performance), it is easy to gain important insights for care monitoring/controlling, quality assurance and the evaluation of the isPO care program for the population enrolled, the network administrators and the stakeholders.

CAPSYS-assist integrates a standardized reporting system that processes the basic data recorded in CAPSYS-docu and converts it into a structured data-based quality report in CAPSYS-assist. The isPO quality report contains various indicators of the quality of the structures (C3) and processes (C4) of the nFC-isPO, which can be used in the context of intra-network quality assurance and cross-network quality improvement measures.

Overall, the standardized reporting system of CAPSYS-assist includes not only the structured data-based quality report but also reports on the local evaluation of health care, contract controlling and accounting.

1. *Evaluation reports:* The reports on the evaluation of local health care are still under development in the current phase of the isPO project. The report will contain data on clinical characteristics of patient care, but also enable statements to be made on the effectiveness of care at the level of individual cases ([145]; initial health effect, etc. (see Fig. 1).
2. *Monitoring reports:* The reports for local monitoring of contractually agreed services provide the participating health insurance companies with information on the services provided for their legally insured patients. These reports are sent to the health insurance companies on a quarterly basis.
3. *Billing reports:* At the current point in time of the isPO project the funding bodies of the IF receive a quarterly invoice for the case rate per care area at steps 0 to 3b (see Fig. 1). If the nFC-isPO were to be transferred to regular health care, these reports will have to be sent to the respective health insurance companies. Given that there are over 100 health insurance companies in Germany, this can only be managed in IT terms.

## Care pathways of the isPO care program

Within the nFC-isPO, the care pathways represent the second central component (C2) of the isPO care program. On the one hand, the nFC-isPO contains the general care pathways, as stipulated in the contract on *"special care*" according to § 140a SCB V (see Fig. 5). On the other hand, there are detailed step-related minor care pathways for each step of the tiered psycho-oncological care program, which cannot be presented in this paper due to their scope.

For each of the minor care pathways at step 0 to 3b a separate algorithm and specific selection and execution recommendations (see Fig. 3) have been developed and will be integrated into CAPSYS-assist during the isPO project.

The formal structure of these pathways [101] is the same via the separate algorithms within CAPSYS-assist. The same applies for the access to the individual path algorithms from CAPSYS-docu, which is designed in such a way that the path algorithm is first accessed from a specific CAPSYS-docu module before the respective recommendations can be accessed. Within the path algorithm, the clinical and/or formal-administrative selection and execution recommendations are displayed according to a button marker that is a graphical representation of a decision or action element of the algorithm [101]. Some of the “*w-elements*” of the execution recommendations are also assigned to specific care documents as pdf files, which contain, for example, the written instructions, evaluation and interpretation of the deployed psychometric instruments of the program, specific service provision instructions taken from the isPO care manuals, or the template of a doctor's letter for demonstration purposes.

Several things can be achieved simultaneously with this structure of care pathways,

1. a contractually appropriate service delivery in the everyday reality of patient care in all care areas,
2. the proof of "*contents, goals and working methods*" as required in the AWMF guideline [44],
3. transparency and monitoring of care and case management over the course of service delivery,
4. a form of *"training on the job"* for newly hired service providers,
5. quality assurance, which refers to the implementation of concrete aspects (recommendations) of the service delivery, and finally
6. targeted change management as a result of decisions for quality improvement or quality development.

### Patient allocation and follow up within the isPO care program

The nFC-isPO is intended to ensure needs-based care for all cancer patients according to the NCP [35]. This is achieved by conducting a psycho-oncological examination of the health care needs of each cancer patient at the beginning (T1, enrolment), during (T2, 4th month after enrolment) and at the end (T3, 12^th^ month)) of patient care within the isPO care program. The examination should be carried out in accordance with the national guideline on psycho-oncological diagnosis, counselling and treatment of adult cancer patients, which requires the use of valid psychometric screening instruments [44].

The psycho-oncological examinations in the isPO care program are carried out with the help of three psychometric instruments,

1. Hospital Anxiety and Depression Scale (HADS [64]),
2. Psychosocial Risk Questionnaire (German: Psychosozialer Risikofragebogen; PSR [65])
3. Cognitive-Emotional Coping with Cancer questionnaire (CECC) (German: Fragebogen zur Kognitiv-Emotionalen Auseinandersetzung mit Krebs; KEA-K) [146].

At the time of enrolment (T1) in the isPO program, the HADS and the PSR are used to establish indications (see Fig. 5) and the CECC is used for treatment planning in the care areas at step 3a and 3b. During the course (T2) and at the end (T3) of psycho-oncological care, the questionnaires are used for re-assessment and outcome evaluation.

Within the isPO care program the baseline examination is performed immediately after the written informed consent at intake (see Table 1, case management, intake; Fig. 5, T1). The threshold values of the HADS and the PSR, determined in Fig. 5, represent the evidence supported allocation criteria to decide whether a patient is offered psychosocial care (step 2), psycho-oncological psychotherapeutic care (step 3a) or complex psycho-oncological care (step 3b). Each patient enrolled in 19 is offered the option of using a contact person in their cancer continuum (step 0) and receiving information from an onco-guide (step 1).

The following psychometrically criteria, as determined by the care concept and the special care contract, apply to the indications for the care areas at step 2, step 3a and step 3b.

*Step 2:* If the emotional distress of a patient is below the threshold value for moderate distress (HADS-T <15 [64]), the patient is considered to experience a low level of emotional dysregulation and does not receive an offer of psycho-oncological-psychotherapeutic care at step 3. However, if there are moderate to high psychosocial problems (PSR ≥3 [65]), the patient is offered psychosocial care.

*Step 3a:* If the emotional distress of a patient is above the threshold value for moderate distress (HADS-T ≥15 [64]), the patient is considered to be experiencing a level of emotional dysregulation that indicates an offer of psycho-oncological psychotherapeutic care.

*Step 3b:* If the emotional distress of a patient is above the threshold value for moderate distress (HADS-T ≥15 [64]), and if the psychosocial problems are above the cut-off point (PSR >5 [65]), the patient is considered to be experiencing elevated levels of emotional and psychosocial distress and is offered the complex form of psycho-oncological care.

The psychometric indication criteria presented are accessible to service providers at various points via the CAPSYS-assist system. Within the care area of "*case management*" they can be looked up in the corresponding selection and execution recommendations of the minor care pathway at step 0, care function *"planning/linking"* (see Table 1).

Allocation criteria for individual care needs are contained in CAPSYS-docu in such a form that, after relevant questionnaire values have been recorded, an automated data evaluation and step allocation takes place. The service providers responsible for a specific care area receive a message in their CAPSYS-docu module informing them that a new patient requires psycho-oncological care in their care area (note: The team of the respective care area decides which colleague is responsible for service delivery in an individual case). Finally, after documentation of the examination results and the corresponding indication in CAPSYS-docu, CAPSYS-assist automatically generates a doctor's letter. The letter will be sent to the attending physician for information about the enrolment, the examination results and the care areas in which the patient receives psycho-oncological care. The attending physician also receives a corresponding doctor's letter after re-assessment in the 4th month (T2) and after the evaluation assessment at the end of the psycho-oncological care in the 12th month (T3).

### Psychometric tools within the isPO care program

*Hospital Anxiety and Depression Scale (HADS)* [64]. The HADS is a self-rating questionnaire assessing anxiety and depression in medical patients. Its use in clinical practice is recommended in psycho-oncological research [147–149]. The Hospital Anxiety and Depression Scale is also recommended in guidelines as one of several screening tools [44, 83, 84], but not as a case-finding instrument [150]. In the German guideline on psycho-oncology of the AWMF, the scale is considered as gold standard [44]. The Hospital Anxiety and Depression Scale comprises of two 7-items subscales assessing anxiety (HADS-A) and depression (HADS-D). Each item is scored on a four-point Likert-type scale (0–3), with item-specific answering formats. After recoding reverse items, item scores are summed up, with higher scores indicating higher levels of anxiety and/or depression. The scores of the individual scales of the HADS can be added to produce a total value (HADS-T), which covers a value range from 0 to 42. A total value of ≥ 15 indicates moderate to high psychological distress [64, 151]. Although caution is advised when using HADS in clinical care [152], and although there is no definite consensus about the optimal overall HADS score to determine the psychological distress of cancer patients, the HADS total score is still considered appropriate for use in clinical practice [148, 153–156]. The HADS total score of ≥ 15 was used in 19 as an indication of moderate to high psychological distress, as suggested by the HADS test authors [64, 150, 151] and by the satisfactory psychometric criteria of that cut-off score [148, 155, 157–160].

*Psychosocial Risk questionnaire (German: Psychosozialer Risikofragebogen; PSR)* [65]. The PSR is also a self-rating questionnaire that was developed for the isPO project. Patients are first asked on a five-point Likert-type scale (1 = *“I can cope alone*” to 5 = “*I urgently need help*”), whether they currently need help to cope with psycho-social challenges due to cancer or cancer treatment, followed by a question assessing on a five-point Likert-type scale (1 = "*I will not need any support*" to 5 = "*I will definitely need support*") on the anticipated health care needs by psycho-social services or facilities within the next months (need for help items). Next, patients can assess their health care needs due to cancer or cancer treatment within the next three months on a four-point Likert-type scale (0 = *"no need for support"* to 3 = *"high need for support")* for 21 different aspects (e.g., *"body care”, "intake of food",* or *"housekeeping*"). The psychometric properties were tested and the PSR was found to be a valid and reliable instrument [65].

*Cognitive-Emotional Coping with Cancer questionnaire* (CECC) (German: Fragebogen zur Kognitiv-Emotionalen Auseinandersetzung mit Krebs; KEA-K) [146]. The CECC is a self-rating questionnaire developed at beginning of the isPO project to measure aspects of emotional regulation with cancer within the last seven days, based on the common-sense model of illness and health [161] and the emotion-regulation model [77]. It comprises of 21 items with a six-point Likert-type scale (0 = “*do not agree at all*” to 5 = “*totally agree*”) per item. The CECC measures the following five facets of emotional regulation regarding cancer: (1) optimism/hope, (2) distraction/suppression, (3) uncontrollable thoughts, (4) cognitive reappraisal, and (5) cognitive avoidance. The CECC revealed a good construct validity, test–retest-reliability, and internal consistency of all five subscales [29].

In 19 the CECC is deployed together with the HADS and the PSR at the three points of assessment at enrolment (T1), during (T2) and at the end (T3) of psycho-oncological care. The questionnaire does not serve the indication at intake, but will provide the psychotherapist in the care area at step 3 information on the specific emotional dysregulation of a patient. It is used to support the service provider in planning and adapting his psycho-oncological care strategy to the specific facets of the emotional regulation of his patient.

At the baseline assessment (T1) a medical history and master data questionnaire is additionally used for formal-administrative purposes of care and care management. This questionnaire assesses basic personal information of the patient (i.e., name, gender, date, and place of birth, marital status, religious confession, nationality, educational background, professional background, and professional status) including contact information (address, phone number, and email), as well as relevant health insurance information.

The procedure for assigning cancer patients to psycho-oncological care, based on a valid psychometric screening procedure, is well established by psycho-oncological research [38, 49, 162, 163], is recommended in international and national guidelines [44, 83–85] and is going to be successively implemented into practice in many institutions in the in-patient and out-patient health sector [38, 57, 99, 109, 164, 165]. However, if a care provider (psychotherapist) decides in care practice that a patient is to be cared for in another care area than the one indicated, this can be recorded as a *“critical incident”* in CAPSYS-docu and a step change can be made. The integration of such a procedure into the organization plan of the care process of cancer centres is still challenging, but even so possible and necessary [30, 166–168].

## Discussion

Due to the high yearly incidence of new cancer cases and their impact on the lives of cancer patients, it is crucial to provide adequate psycho-oncological care to all those in need. Based on these facts, the National Cancer Plan of the Federal Government of Germany from 2008 therefore demanded that *"all cancer patients receive appropriate psycho-oncological care when needed"* [35]. Yet psycho-oncological care is still far from being available to all cancer patients in Germany. In order to achieve this important goal, the NCP demands that new forms of psycho-oncological care be developed and integrated into the in-patient and out-patient structures of cancer care, and that a sustained high quality psycho-oncological patient care must be guaranteed.

By funding the project "*integrated, cross-sectoral psycho-oncology*" (isPO), the Innovation Fund (IF) of the Joint Federal Committee of Germany hopes to create an innovative form of care (nFC-isPO) that can be offered nationwide within the legal framework of the Social Code Book V for the statutory health insurance funds [60, 61]. The independent institute IMVR (Institute for Medical Sociology, Health Services Research and Rehabilitation Science, University of Cologne) was commissioned by the IF to comprehensively evaluate the nFC-isPO and the isPO care program [69].

Obviously, especially when considering the six separate components, the nFC-isPO is a highly complex form of care. The same applies to the isPO care program with its complex, closely intertwined elements of the care concept and the care pathways. And finally, the linking of the domains of care and the core service functions (see Table 1) with the related operational recommendations (see Fig. 3), the documentation modules of CAPSYS-docu and the databased quality reports of CAPSYS-assist also represents a very complex system.

This raises the simple question, "*Is it worth the effort?*" There is a simple answer to this, "*yes, due to the complexity of health care in any, but especially in the German health care system, this effort is indispensable!*”.

Complexity is an essential characteristic of the day-to-day patient care in health care organizations of a given health care system within a particular country. All health care systems are highly complex systems that cannot be reduced to easily controllable conditions, but are self-organized and self-regulating systems [74, 169–174]. The structure of the German health care system is considered a particularly complex and therefore not easy to understand self-regulating system [58, 59, 175]. Actors in the field of this health care system include associations and interest groups representing the various providers and occupational groups, health insurance companies, quality assurance institutions, the Ministry of Health, as well as patient organizations and self-help groups [59]. And in Germany´s statutory health insurance, corporatist bodies form the self-governing structures determining the provision and financing of health care services, with the Federal Joint Committee being the most important decision-making body [176]. The German Social Code Book V for statutory health insurance funds (SCB V) provides the regulatory framework. According to the Innovation Fund (IF) of Germany's Federal Joint Committee, the isPO project has been prompted to develop and implement the nFC-isPO in such a way that it fits into this complex SCB V framework of the healthcare system.

The complexity of the German health care system is immediately apparent when one considers § 70 SCB V on “*Quality, Humanity and Economy*”, which states in the first paragraph:

1. The health insurance funds and the service providers shall ensure that the care of the insured persons is able to meet their needs, is uniformly provided and in accordance with the generally recognized state of medical knowledge (defined here as clinical requirement).
2. The care of the insured persons must be sufficient and expedient, must not exceed what is necessary (defined here as technical requirement),
3. must be provided in the professionally required quality (defined here as quality requirement), and
4. must be provided economically (defined here as economical requirement).

These legal requirements of § 70 SCB V alone necessarily make nFC-isPO a highly complex form of care. Nevertheless, as this is the German context, the nFC-isPO with its six components (see Fig. 1), is designed to meet all four requirements.

1. *Clinical requirements*: The care concept (C1) with the need-based stepped care approach reflects the “*state of the art”*, and the care pathways (C2) with the selection and execution requirements represents, that patient care can be *“uniformly*” and equally provided to cancer patients.
2. *Technical requirement*: firstly, a consistent transfer of the clinical care concept and the formal-administrative requirements of the special care contract into the recommendations of the care pathways characterize the nFC-isPO. Thus, for the population of cancer patients and at the level of the individual cancer patient, it is established what is considered “*necessary*” for the psycho-oncological care of cancer in nFC-isPO.

Secondly, aligning psycho-oncological care with predefined standards, aims or outcomes (see Fig. 3) is central from an entrepreneurial point of view, from the point of view of stakeholders and from the patient's perspective. Both, the outputs of a given organizational structure (C3) and of a given service process (C4) are important, as well as the central goal of all measures, the patient-oriented results of each single patient care, respectively the outcome quality of nFC-isPO as such. To the extent that the outputs and outcomes of nFC-isPO are based on valid clinical and formal-administrative recommendations, statements can be made as to whether the given patient care is “*expedient”*. Although the nFC-isPO developed in the isPO project is not yet based on such valid data, it provides a system in which this validity can be created.

Thirdly, the clinical and formal-administrative recommendations provide the basis for the construction of the IT-system CAPSYS (C5) with its interwoven parts CAPSYS-docu and CAPSYS-assist. This makes it possible to check and ensure that each patient has received the services necessary for him/her in “*sufficient*” quantity, both in terms of the "*right*" services and the "*right*" quantity of services.

3) *Quality requirement*: The German Social Code Book V (SCB V) demands that care provided is of the required professional quality. This in turn requires that the professional quality of care is known and defined, but above all that it is reflected in the structures of the health care organization and the processes of patient care. With the specific care functions of each care area of the isPO care program (see Table 1) and the operational recommendations of the care pathways (see Fig. 3), the structural and procedural “*quality characteristics of professional patient care”* are defined according to the nFC-isPO. In isPO, the audit is carried out via the data-based reporting system and the network's internal quality circles and the improvement via the cross-network quality workshops (C6).
4) *Economic requirements*: The demand that health care organizations operate economically at the level of the population and the individual level means, at least in the German health care system, that they neither provide too much, nor too little, nor provide the wrong care, which in turn is based on knowing what is the "*right*" care to provide. The nFC-isPO explicitly specifies what it regards as "*doing the right thing*" and "*doing the right thing right*" (see Fig. 3 and Table 1) and thus makes all its measures potentially available for economic evaluation.

Overall, the nFC-isPO is based on the fundamental idea that health care must be "*rational*", i.e. it should be based on transparent, reasonable and targeted knowledge and action. To the best of the authors' knowledge, to date there are no population-based health care programs in psycho-oncology, either in Germany or internationally, that take equal accounts of the state of knowledge in psycho-oncology, aspects of professionally required quality, and economically service provision as those provided by the isPO care program of the nFC-isPO. Nevertheless, in psycho-oncology, population-based care programs are increasingly called for [38, 91] and the elements of which these programs should consist seem to be comparable to the elements formulated in the SCB V.

In 2020, the Board of Directors and Research Committee of the American Psychosocial Oncology Society (APOS) selected 10 APOS members with expertise in care delivery models [38]. The task force was established to develop recommendations for a framework for planning the delivery of psychosocial oncology as part of a Delphi process. The task force formulated three principles according to which care delivery models should be developed and put into practice in patient care [38]:

Principle 1: Population-based approaches to care delivery.

Principle 2: Parameters that shape psychosocial oncology services.

Principle 3: High-quality psychosocial oncology care across all components of care.

### Principle 1

According to the APOS task force [38] an ideal model of care would pursue a population-based approach, allowing.

 

- prompt health care needs identification throughout the cancer trajectory;
- provision of right services, at the right time, in the right place, by the right provider;
- the tracking of cancer and supportive care outputs (e.g. resource utilization) and outcomes (e.g. quality of life);
- rapid intervention and treatment course adjustment.

In order to achieve these goals, the model requires approaches such as case management based stepped-care and/or cooperative care models [38]. According to the authors, these elements of a population-based approach are included in the care concept (C1) and care pathways (C2) of the nFC-isPO (see chapter: *the isPO care program*).

### Principle 2

 According to the APOS task force [38] three interdependent parameters shape psychosocial oncology services: (a) resources (funding, staffing, technology, and space); (b) aims (breadth and depth of services); and (c) scope (target population). The nFC-isPO and the corresponding contracts for this special form of patient care attempt to take these aspects into account as follows:

*Funding: *During the project period the nFC-isPO is funded by the IF and could afterwards be covered by the statutory health insurance funds.

*Staffing:* The nFC-isPO specifies the number and position of heads, managers and staff members in the organization chart of psycho-oncological care networks (C3). The calculation of staff resources results from the specified and contractually agreed numbers of services within 12 months of patient care (see Table 1).

*Technology and space:* The psycho-oncological care organization (C3) is responsible for the technical equipment, including the CAPSYS (C5) IT-system, the premises and other structural requirements in accordance with the isPO cooperation agreement.

### Principle 3

According to the APOS task force [38] it is recommended, that cancer care centres should strive to ensure the delivery of high quality psychosocial oncology care across all components of care. The issue of providing high quality psychosocial oncological care is the main topic of the isPO project (see chapter: *the isPO care program*). However, no valid statements can be made at this stage of the project regarding the quality of patient care in the isPO care program. But the reader can certainly judge from the structure of nFC-isPO whether the quality of psycho-oncological patient care can be evaluated sufficiently well with the quality assurance and improvement measures of the nFC-isPO (C6).

In addition to the three principles the APOS task force generated 8 elements of “*aims and scope of services*”, 6 elements of “*identifying needs and linking services*” and 6 elements of “*safeguards for adequate treatment and follow-up and quality oversight* “ and ordered them into tiers of successively more aspirational practices. The authors cannot comment on these elements in detail in the context of this work. However, these elements have also been taken into account in the development of the nFC-isPO and will be included in the external evaluation of the isPO project by the IMVR.

The four-year isPO project was selected by the IF as particularly worthy of funding because it is considered suitable for fulfilling the requirements of the Federal Government's National Cancer Plan (NCP) to further develop the existing "*oncological care structures and quality assurance*" in the psycho-oncological field.

The subject of this paper was to demonstrate this and illustrate how the nFC-isPO developed within the framework of the isPO project and implemented in four local psycho-oncological networks in the federal state of North Rhine-Westphalia in Germany can meet the legal requirements of SCB V. The authors are convinced that the nFC-isPO meets the current national and international requirements for the planning and provision of high-quality psycho-oncological services as required by the SCB V and as presented by the APOS task force.

## Challenges and limitations

As with any complex health care program, the nFC-isPO is also subject to a number of limitations and the nFC-isPO would face challenges before it can be transferred and disseminated into the regular statutory health care system in Germany.

Firstly, there are limitations resulting from the isPO project that relate to the development of nFC-isPO and its implementation. Even if the project with a four-year duration seemed to offer enough time to develop, implement and evaluate the nFC-isPO, from the current perspective the approved project duration has to be considered too short, as the one-year development period alone was not enough to create a care model fully developed in all components. Although it was planned to revise the prototype of the nFC-isPO during the first year of its implementation under practical conditions and to make use of the results of the quality improvement and the formative process evaluation, even the second project year was not sufficient to produce the final version of the nFC-isPO, as the implementation of the prototype in real-world practice required considerable fine-tuning, which went beyond the planned first phase of internal optimization and the scheduled first formative evaluation by the IMVR. It is expected that the nFC-isPO will remain subject to constant change as part of the continuous quality improvement (C6) integrated into it, even if the project version of the nFC-isPO will be submitted to the IF at the end of the isPO project.

At this point, further limitations should also be mentioned, such as.

1. the project-related restrictions due to the enrolment criteria (newly diagnosed cancer patients with a good 1-year prognosis),
2. the considerable formalities that the patients have to fulfil at the beginning of enrolment (declaration of participation in the special care contract and agreement to participate in the isPO study),
3. the interface between the referring physicians (oncologists) and the case managers on the psycho-oncology side was only established in the project, which meant that not all potential patients could be reached, and
4. the fact that the nFC-isPO can only be offered to those patients with statutory health insurance whose health insurance fund participates in the special care contract.

The first two restrictions are only relevant for as long as the isPO project lasts, after which isPO can be offered to all cancer patients and study-related formalities will be eliminated. The third point, on the other hand, is of great relevance, since patients cannot be enrolled in isPO without being assigned to it by the treating physician. If one considers the general study situation regarding the significance of the medical recommendations for psycho-oncological care, it becomes clear that the willingness of patients to participate in psycho-oncological care increases considerably with good medical information and recommendations [52, 176, 177]. Although much has been done during the implementation of the nFC-isPO to optimize the horizontal cooperation and coordination between treating physicians and isPO case management, the authors assume that these efforts will also be a constant challenge of isPO in regular care. The fourth aspect is actually not a limitation of the nFC-isPO, but of the contract for special care under Section 140a SCB V. This stipulates that the special care contract can only be offered to those patients whose insurance fund has signed the contract.

Secondly, there are limitations due to the design of the nFC-isPO itself. As already mentioned, the isPO care program is designed for newly diagnosed cancer patients, so that the results of the external evaluation cannot be transferred to all cancer patients. We are convinced of its value and therefore recommend that in the future the nFC-isPO may include the psycho-oncological care of all cancer patients, regardless of whether they are newly diagnosed or in advanced stages of the disease and independent of an early phase of cancer therapy.

Another factor that may be considered a limitation is that any psycho-oncological care network, however small, must meet the same legal and contractual requirements and the network internal management requirements, e.g. keeping the isPO care program at a state of the art or at recommended quality levels; internal quality management; reporting obligations; accounting. But even if many local networks wanted to participate in the nFC-isPO, they would all have to fulfil the same tasks of establishing a psycho-oncological care network and the sustainable, quality-assured implementation of the nFC-isPO. Since the project sponsor IF has demanded that a new form of care is developed that can be transferred to the regular supply according to SCB V, the consortial partners have already integrated a central network management system into the isPO project responsible for network support and cross-network quality improvement. Therefore, if a central office for cross-network isPO management were to be established, it could support both external actors (e.g. the statutory health insurance funds) and the management and service providers in the local psycho-oncological networks with important tasks and duties. The central isPO administration should act as an intermediary between the health insurance funds and the networks and ensure that all contractually agreed tasks, obligations and services are implemented as planned. Moreover, it could support the local networks in meeting the demands (e.g. from SCB V, NCPs and the national guideline) and in keeping patient care up to date with the state of the art in psycho-oncology. A central administration may also support the networks in accomplishing the legal obligation for quality assurance and continuous quality improvement, as well as for reporting and accounting with the health insurance companies. And finally, it could facilitate in-house training and qualification courses or offer support for questions regarding the supply contract, problems in service provision or malfunctions of the CAPSYS IT-system (e.g. like a first level help desk or second level IT-support), and also in-house measures of health care improvement.

Should, after the four-year funding, the external summative evaluation of the IMVR come to a positive result and if therefore the IF agrees to a Germany-wide transfer of the isPO approach, new challenges will arise. It does not seem realistic to implement the isPO approach straight forwardly throughout Germany (geographical coverage). However, the structure of the nFC-isPO is designed for successive dissemination into the health care system. In the first step, cancer centres and physicians in private practice join together to form local psycho-oncological networks, cooperate with health insurance companies and a central management office. In the second step, the local networks are supported in setting up nFC-isPO, instructed in the isPO care program and familiarized with the CAPSYS system. At the same time, in a third step, the care contract is concluded and the cooperation agreement signed and immediately afterwards the new form of patient care can begin. This entire process can take place very quickly, since with the nFC-isPO all components are already available to allow psycho-oncological care of cancer patients at a high level of quality and in accordance with the provisions of SCB V. The authors now have more than two years of experience in the development, refinement and implementation of the nFC-isPO approach and it appears to be working.

Our conclusion is that the nFC-isPO can be implemented within the framework of the Social Code Book five (SCB V) for statutory health insurance, as it has been running since the January of 2019 on the basis of the "*special care*" contract pursuant to Sect. 140a SCB V.

## Data Availability

The data sets generated and/or analyzed in the current study are not publicly available, due this paper is not based on study data but represents methodological work, but the data from the mentioned isPOstudy are available from the corresponding author on request as soon as the isPO project is completed.

## Footnotes

1 After the successful completion of the isPO project, this procedure should enable those responsible in the German health care system to determine in advance the organizational structure, the necessary financial and technical resources, the qualification requirements of the staff, or the coordination and management of care before the decision is made to transfer the nFC-isPO to the German health care system.
